# A Successful National and Multipartner Approach to Increase Immunization Coverage: The Democratic Republic of Congo Mashako Plan 2018–2020

**DOI:** 10.9745/GHSP-D-22-00326

**Published:** 2023-04-28

**Authors:** Paul Lame, Augustin Milabyo, Sylvia Tangney, Gloire O. Mbaka, Christophe Luhata, Jean-Bernard Le Gargasson, Christelle Mputu, Nicole A. Hoff, Sydney Merritt, Dalau M. Nkamba, Djariatou Sow Sall, John Samuel Otomba, Amine El Mourid, Paul Lusamba, Kamel Senouci, Emmanuel Bor, Anne W. Rimoin, Didine Kaba, Guillaume Mwamba, Elisabeth Mukamba

**Affiliations:** a Expanded Programme on Immunization, Ministry of Health, Kinshasa, Democratic Republic of the Congo.; b Department of Epidemiology, Fielding School of Public Health, University of California Los Angeles, Los Angeles, CA, USA.; c UCLA-DRC Health Research and Training Program, University of California Los Angeles, Kinshasa, Democratic Republic of the Congo.; d Gavi, the Vaccine Alliance, Geneva, Switzerland.; e Acasus, Tsim Sha Tsui, Hong Kong.; f Kinshasa School of Public Health, University of Kinshasa, Kinshasa, Democratic Republic of the Congo.; g UNICEF Headquarters, New York, NY, USA.; h World Health Organization, Geneva, Switzerland.; i Bill & Melinda Gates Foundation, London, United Kingdom.; j VillageReach DRC, Kinshasa, Democratic Republic of the Congo.

## Abstract

**Background:**

The immunization system in the Democratic Republic of the Congo faces many challenges, including persistent large-scale outbreaks of polio, measles, and yellow fever; a large number of unvaccinated children for all antigens; minimal and delayed funding; and poor use of immunization data at all levels. In response, the Expanded Programme on Immunization within the Ministry of Health (MOH) collaborated with global partners to develop a revitalization strategy for the routine immunization (RI) system called the Mashako Plan.

**Mashako Plan Design and Development:**

The Mashako Plan aimed to increase full immunization coverage in children aged 12–23 months by 15 percentage points overall in 9 of 26 provinces within 18 months of implementation. In 2018, we conducted a diagnostic review and identified gaps in coordination, service delivery, vaccine availability, real-time monitoring, and evaluation as key areas for intervention to improve the RI system. Five interventions were then implemented in the 9 identified provinces.

**Discussion:**

According to the 2020 vaccine coverage survey, full immunization coverage increased to 56.4%, and Penta3/DTP3 increased to 71.1% across the Mashako Plan provinces; the initial objective of the plan was reached and additional improvements in key service delivery indicators had been achieved. Increases in immunization sessions held per month, national stock of pentavalent vaccine, and supervision visits conducted demonstrate that simple, measurable changes at all levels can quickly improve immunization systems. Despite short-term improvements in all indicators tracked, challenges remain in vaccine availability, regular funding of immunization activities, systematic provision of immunization services, and ensuring long-term sustainability.

**Conclusions:**

Strong commitment of MOH staff combined with partner involvement enabled the improvement of the entire system. A simple set of interventions and indicators focused the energy of managers on discrete actions to improve outcomes. Further exploration of the results is necessary to determine the long-term impact and generate all-level engagement for sustainable success in all provinces.

## Abstract (precis)

An innovative rapid revitalization strategy strengthened routine immunization systems in the Democratic Republic of the Congo through the engagement of subnational governments and could be applied to other low- and middle-income countries.

## Teaser

**Key Findings:**

- The strategy to strengthen routine immunization (RI) placed particular emphasis on service delivery, management, real-time monitoring, evaluation, and vaccine availability.
- Existing RI programs can be rapidly strengthened using simple metrics and straightforward interventions.
- Subnational government engagement is important to create ownership and collaboration amongst stakeholders.

**Key Implication:**

- As new vaccines are added to RI schedules, the processes identified during the Mashako Plan implementation will continue to inform practices that ensure the strength of immunization systems in the DRC and may be useful to other low-and-middle income countries.

## BACKGROUND

Immunization is a highly cost-effective public health intervention that leads to a reduction in global child morbidity and mortality, with approximately 3 million child deaths prevented each year.^1^^,^^2^ After the success of the smallpox eradication program, the Expanded Programme on Immunization (EPI) was launched in 1974 to ensure that all children globally had access to vaccines.^3^ The routine immunization (RI) system is meant to ensure that children are fully vaccinated against vaccine-preventable diseases by ensuring the sustainable, reliable, and timely delivery of vaccines to children by health care workers.^4^ RI system vaccines vary by country but generally include bacillus Calmette-Guérin; hepatitis B; polio; diphtheria, tetanus toxoid, and pertussis-containing vaccine (DTP-containing vaccine); pneumococcal; *Haemophilus influenzae* type b; rotavirus; and measles-containing vaccines (Table 1).^5^

**TABLE 1.** Routine Immunization Schedule in the Democratic Republic of the Congo^12^

Vaccine	Description	Schedule
BCG	Bacillus Calmette–Guérin vaccine	Birth
Penta3	Diphtheria, tetanus, pertussis, *Haemophilus influenzae* type b, and hepatitis B vaccine	6, 10, 14 weeks
OPV	Oral polio vaccine	6, 10, 14 weeks
Pneumo	Pneumococcal conjugate vaccine	6, 10, 14 weeks
IPV	Inactivated polio vaccine	14 weeks
Measles	Measles vaccine	9 months
YF	Yellow fever vaccine	9 months
Rota	Rotavirus vaccine	6, 10, 14 weeks

Despite widespread access to vaccination services, the burden of vaccine-preventable diseases in low- and middle-income countries (LMICs) remains high, accounting for 1.5 million preventable deaths.^6^ Despite the ambitious goals set by the Global Vaccine Action Plan in 2010 (90% coverage for all antigens nationally, 80% coverage in every district), progress has plateaued in the past decade, particularly in LMICs.^6^^,^^7^

In the Democratic Republic of the Congo (DRC), there are significant logistical challenges to the implementation of an effective RI program. In addition to the size of the country and ongoing security issues in the east, the population is steadily growing due to high fertility, with 3.2 million surviving children younger than 1 year in 2018 and a national birth cohort increasing by 100,000–150,000 children every year.^8^ The health system in the DRC is decentralized with different responsibilities falling to the 6 different levels of health care delivery: national, provincial, district (or “antenne” in the EPI vaccine delivery program structure), health zone, health area, and health facility.

There are significant logistical challenges to the implementation of an effective RI program in the DRC.

Between 2014 and 2018, a drop in coverage of all antigens measured in nationally representative surveys occurred. The Demographic and Health Survey (DHS) in 2014 showed coverage of DTP3 was 60.5% in children aged 12–23 months.^9^ Yet, the Multiple Indicator Cluster Survey (MICS) in 2018 measured coverage of the third dose of the pentavalent vaccine (penta3)—a DTP-containing vaccine that also includes hepatitis B and *Haemophilus influenzae* type b antigens—and reported 46.2% penta3 coverage in the DRC, among children aged 12–23 months.^9^^,^^10^ It is important to note that these results from the 2017–2018 MICS report have been shown to be an outlier, reporting lower coverage than expected.^8^ DTP3/penta3 is often used as an indicator antigen to estimate performance of the RI system.^11^ A similar drop in coverage was observed among fully immunized children, defined as those who have received 1 dose each of bacillus Calmette-Guérin and measles vaccine and 3 doses each of a DTP-containing vaccine (i.e., DTP3 or penta3) and oral poliovirus vaccine (Table 2).^12^ Coverage of fully immunized children was consistently low according to national surveys, with the 2014 DHS reporting 45.3% full immunization coverage and the 2018 MICS reporting 35.0% full immunization coverage (Table 3).^9^^,^^10^

**TABLE 2.** DTP3/Penta3 Coverage in Initial Mashako Plan Provinces, Democratic Republic of the Congo^9^^,^^10^^,^^25^^–^^27^

**Province (Former Province Name)**	**Coverage Survey Conducted**				
	**DHS 2013–2014,^a^ %**	**MICS 2017–2018, %**	**Baseline VCS 2018–2019, %**	**VCS 2020, %**	**VCS 2021–2022, %**
Mongala (Equateur)	42.6	16.7	N/A^b^	37.1	35.9
Tshuapa (Equateur)	42.6	26.5	N/A^b^	42.9	16.1
Haut Katanga (Katanga)	51.3	60.7	N/A^b^	67.7	61.3
Tanganyika (Katanga)	51.3	25.2	60.8	63.2	32.0
Haut Lomami (Katanga)	51.3	50.4	50.4	77.9	94.5
Ituri (Orientale)	46.0	50.3	N/A	67.7	62.5
Kinshasa	83.7	59.8	70	85.2	88.9
Kwilu (Bandundu)	61.9	25.1	66	86.0	73.6
Kasaï (Kasai-Occidental)	56.2	23.2	51.5	65.6	45.3
5 Mashako Plan provinces with baseline VCS^c^	65.3	41.0	61.5	78.1	72.6
All Mashako Plan provinces	58.4	42.7	N/A^d^	71.1	64.7

Abbreviations: DHS, Demographic and Health Survey; DTP3, diphtheria, tetanus, pertussis; MICS, Multiple Indicator Cluster Survey; N/A, not available; Penta3, pentavalent3; VCS, vaccination coverage survey.

a DHS 2013–2014 was conducted before the division of new provinces in 2015.

b Baseline survey not conducted.

c Does not include estimates from Ituri, Haut Katanga, Mongala, or Tshuapa.

d Missing baseline VCS 2018–2019 data from Ituri, Haut Katanga, Mongala, or Tshuapa.

**TABLE 3.** Full Immunization Coverage in Initial Mashako Plan Provinces, Democratic Republic of the Congo^9^^,^^10^^,^^25^^–^^27^

**Province (Former Province Name)**	**Coverage Survey Conducted**				
	**DHS 2013–2014** ^a^	**MICS 2017–2018, %**	**Baseline VCS 2018–2019, %**	**VCS 2020, %**	**VCS 2021–2022, %**
Mongala (Equateur)	N/A	8.2	N/A^b^	26.1	21.1
Tshuapa (Equateur)	N/A	15.4	N/A^b^	35.1	11.1
Haut Katanga (Katanga)	N/A	45.5	N/A^b^	48.9	44.0
Tanganyika (Katanga)	N/A	21.2	51.7	46.3	13.9
Haut Lomami (Katanga)	N/A	35.7	39.0	69.4	88.9
Ituri (Orientale)	N/A	40.0	N/A^b^	47.9	39.7
Kinshasa	N/A	41.9	61.2	72.8	74.8
Kwilu (Bandundu)	N/A	14.3	50.7	66.2	56.5
Kasaï (Kasai-Occidental)	N/A	13.9	38.2	53.5	30.3
5 Mashako Plan provinces with baseline VCS^c^	N/A	28.1	50.2	64.5	58.7
All Mashako Plan provinces	N/A	30.3	N/A^d^	56.4	49.0

Abbreviations: DHS, Demographic and Health Survey; MICS, Multiple Indicator Cluster Survey; N/A, not available; VCS, vaccination coverage survey.

a DHS 2013–2014 conducted before division of new provinces in 2015, data removed because full immunization values included only 5 antigens until 2017 when this was increased to 7 antigens.

b Baseline survey not conducted.

c Does not include estimates from Ituri, Haut Katanga, Mongala, or Tshuapa.

d Missing baseline VCS 2018–2019 from Ituri, Haut Katanga, Mongala, or Tshuapa.

Based on the MICS survey findings, at least 2.5 million children were estimated to be underimmunized (i.e., had only received some of the routine schedule of vaccines) or nonimmunized (i.e., had not received any routine schedule vaccines) annually (Table 1).^10^ The occurrence of vaccine-preventable disease outbreaks—including multiple, independent emergences of vaccine-derived poliovirus type 2 and continuous large-scale outbreaks of measles—further confirmed this assessment.^13^ In 2019, the largest reported measles epidemic occurred in the DRC, with more than 300,000 suspected cases and 6,000 deaths.^14^ These outbreaks and results from national surveys demonstrated that despite large investments from the government and international donors, there was no significant improvement made in vaccination coverage between 2013 and 2018.

In response to these stagnant vaccination coverage rates, the DRC EPI and its partners developed an emergency plan for the revitalization of RI. This plan aimed to rapidly boost childhood immunization coverage from June 2018 with the vision that “routine is the new emergency.” The plan was named in honor of former Minister of Health Leonard Mashako Mamba, who signed the first support request to Gavi, the Vaccine Alliance (Gavi) for a new vaccine introduction (yellow fever) in 2002 and was a strong proponent of RI and polio eradication. The Mashako Plan used simple steps to target specific challenges in vaccine availability, delivery, and accessibility that could be executed at all levels of the RI system.

The Mashako Plan used simple steps to target specific challenges in vaccine availability, delivery, and accessibility at all levels of the RI system.

## MASHAKO PLAN DESIGN AND DEVELOPMENT

### Diagnostic Review

**FIGURE 1:**
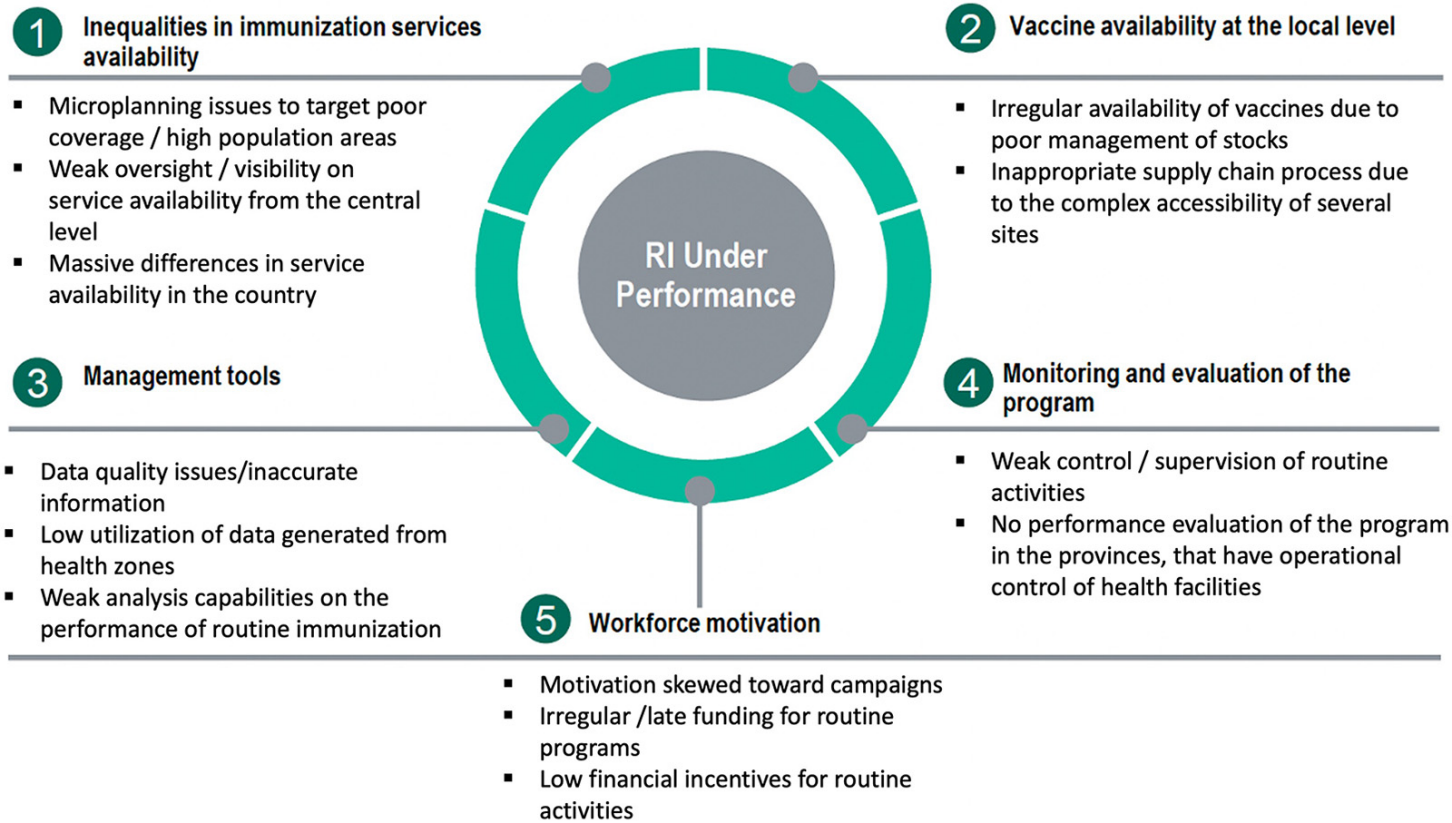
2018 Diagnostic Review Findings Related to the Underperformance of the Routine Immunization System in the DRC Abbreviations: DRC, Democratic Republic of the Congo; RI, routine immunization.

To develop the Mashako Plan, the EPI within the Ministry of Health (MOH), with support from Acasus, Gavi, UNICEF, the Bill & Melinda Gates Foundation (BMGF), and the World Health Organization (WHO), conducted a diagnostic review in March–April 2018. The review aimed to ascertain the most pressing issues and identify vaccine availability and service accessibility. The EPI and partners used available literature, surveys, and administrative data from the District Vaccination Data Management Tool and DHIS2 to conduct a diagnostic review guided by 4 essential themes (Figure 1): (1) service delivery; (2) demand; (3) program management, monitoring, and evaluation; and (4) data quality.

The National Health Development Plan (2016–2020) revealed that among 75% of all facilities that offered vaccination, only 42% had the required equipment or personnel available on the assessment day.^15^ From 2018 DHIS2 data, 36% of all health areas in the country reported organizing less than 1 immunization session (scheduled time for providing RI to children aged younger than 1 year) per week or fewer than 4 per month, while only 10% organized more than 2 per week (more than 8 per month). These numbers indicated a challenge with the regularity and availability of RI services in large portions of the country. Further, the District Vaccination Data Management Tool data revealed that in 2017, 92% of health zones experienced a stock-out of at least 1 antigen. On average, these health zones experienced 7 stock-outs per year for any of the antigens required to fully immunize a child. These stock-outs indicated severe vaccine management issues at all levels that impact the availability of vaccines in health facilities and at immunization sessions.

The 2014 DHS survey data suggest that only 5% of children were vaccine-naïve (zero-dose), which demonstrates that strong demand for routine vaccines existed in the country. More recent interviews with the national, provincial, and health zone program managers indicated that demand for vaccination services from parents remained strong during RI service delivery, with anecdotal evidence of vaccine refusals only during polio mass vaccination campaigns.^16^

More than 7,000 monitoring meetings and 100,000 health facility supervisions were conducted annually to improve program outcomes. While these management routines were available at all levels, there were limited processes enabling provincial and health zone managers to take decisive action on issues at the zonal or provincial levels because the meetings were mostly used to compile and send administrative data to the national level. Granular data from health areas were limited, and health facilities focused mostly on reporting the number of children immunized and meetings held while disregarding measurable and actionable indicators addressing challenges at the facility. In addition, the data collected at the health facility level were difficult to validate, and their accuracy was unknown. Vaccination data from the District Vaccination Data Management Tool were only available at the provincial and national levels 1 to 2 months after activities were completed, which created a lag for decisive action. These evaluations collectively indicated that the EPI did not have the proper tools at its disposal to effectively manage the program and monitor performance at a granular level.

Finally, there were major issues in data quality with very high vaccine coverage reported by administrative systems. The country’s administrative coverage estimates reported an increase of 54 percentage points for DTP3-containing vaccine coverage between 2000 and 2017 to reach 94% coverage and a 46 percentage-point increase in measles coverage in the same years to reach 92% coverage.^17^^–^^19^ These data did not match the survey estimates and were contradicted by the occurrence of vaccine-preventable outbreaks.

### Development and Launch of the Mashako Plan

**FIGURE 2:**
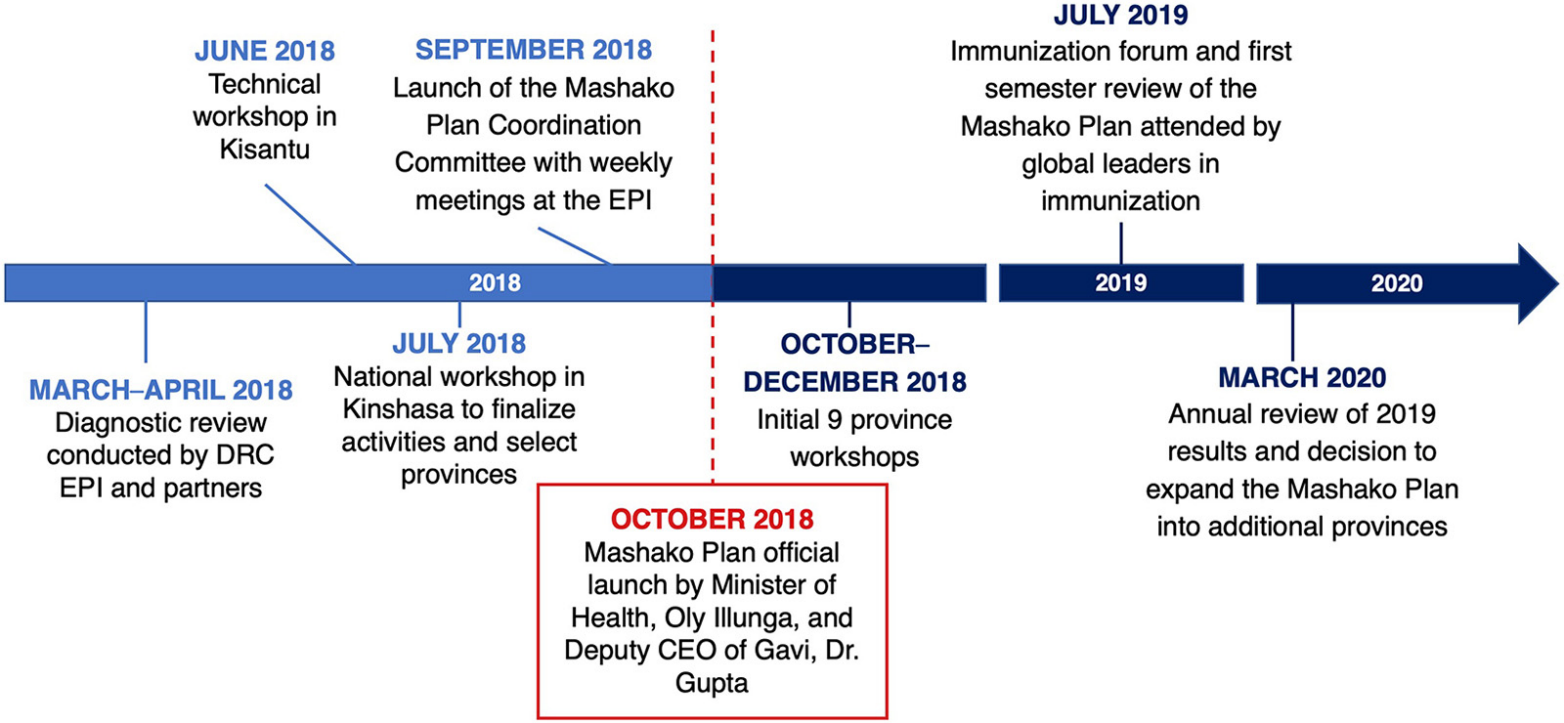
Mashako Plan Implementation Timeline Abbreviations: DRC, Democratic Republic of the Congo; EPI, Expanded Programme on Immunization; Gavi, Gavi, the Vaccine Alliance.

Based on the diagnostic review, the MOH, Gavi, BMGF, and other partners determined that the strong programmatic leadership, available resources, and available technical assistance from partner organizations created a unique opportunity to rapidly improve vaccination coverage with an ambitious time-limited plan (Figure 2). The plan was based on the theory of service delivery, in which stakeholders, including government agencies, identify challenges and establish an implementation plan and a delivery unit to address barriers in service delivery.^20^ The diagnostic review identified barriers in both service delivery and program management. The Mashako Plan was developed to remove these barriers using an evidence-based approach with measurable outcomes.

The diagnostic review of the immunization program identified barriers in service delivery and program management.

In June 2018, based on the results of the diagnostic review, technical workshops were convened to identify priority provinces and key activities to be conducted in collaboration with technical partners (Acasus; BMGF; Gavi; JSI; PATH; University of California, Los Angeles; UNICEF; Village Reach; and WHO). These technical workshops were followed by a national workshop in July 2018, led by the Minister of Health, to validate the plan. In September 2018, national and provincial coordination teams were established to monitor and track the implementation of the plan.

The Mashako Plan was officially launched in October 2018. The initial 9 provinces of Kinshasa, Haut Katanga, Kwilu, Kasai, Ituri, Mongala, Tshuapa, Tanganyika, and Haut Lomami were selected because they were the most populous, contained 45% of underimmunized children in the DRC, reported frequent stock-outs, and were identified as the most vulnerable.^10^ Workshops were held in the initial 9 provinces selected between October and December of 2018.

The Mashako Plan attempted to implement simple procedures and standards, such as minimum number of vaccination sessions per month and supervision visits, that could be executed at all levels of the RI system. These simple activities focused on 5 areas of support for the RI system: (1) coordination, (2) service delivery, (3) vaccine availability, (4) real-time monitoring, and (5) evaluation. It was especially important to ensure that there was support for the plan at all levels of implementation, from the most local level (e.g., vaccinators) to district and provincial management and up to the national level. Instead of attempting to address all RI problems, the Mashako Plan aimed to shift the focus of the immunization program from policy discussion to the implementation of a limited set of concrete activities (described later) that focus on key vaccine performance issues. The plan provided actionable targets to improve the RI system, such as increasing the number of completed vaccination sessions by at least 20% within 18 months of implementation and introducing an annual independent survey to evaluate performance.

## MASHAKO PLAN GOALS AND IMPLEMENTATION

The main goal of the Mashako Plan was to increase full immunization coverage by 15 percentage points in the 9 initial provinces within 18 months of implementation, compared to the MICS 2018 immunization rates, where full immunization coverage was estimated to be 35% for children aged 12–23 months.

The strong collaboration between the DRC government and partners was a critical element in the successful implementation of the Mashako Plan. The overall government contributions to immunization included the payment of salaries of health care workers (nurses and health zone managers) and funding for traditional vaccines with cofinancing from Gavi.

**FIGURE 3:**
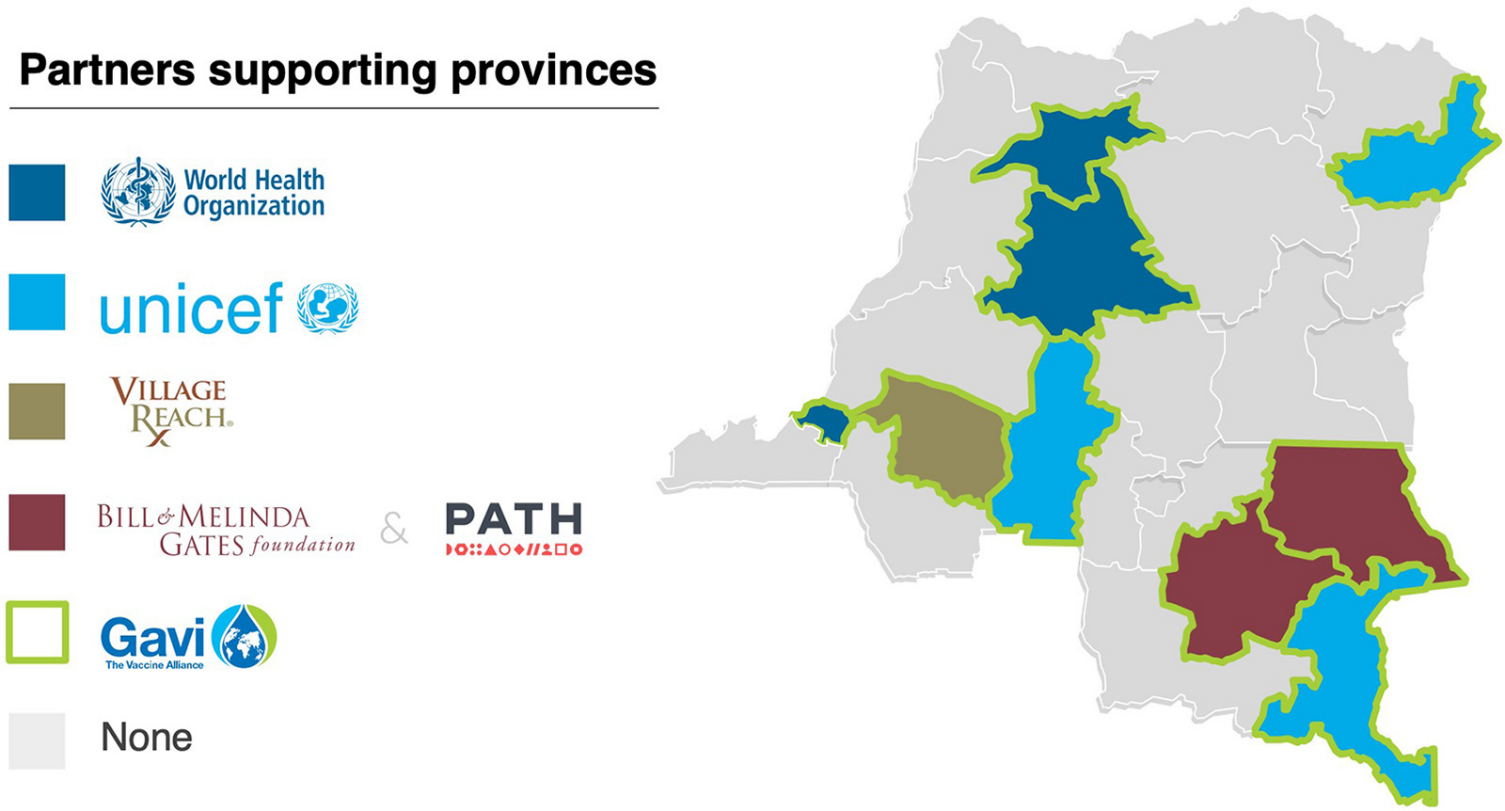
Mashako Plan Provinces

The initial 9 province activities were financed by Gavi, UNICEF, and BMGF through a variety of partnership models. Each of the initial Mashako provinces received financial support from donors for operational activities (Figure 3). Kinshasa, Kwilu, Mongala, and Tshuapa were financially supported by Gavi through the Health System Strengthening grant model implemented by WHO and VillageReach.^21^ Kasai, Ituri, and Haut-Katanga were supported by UNICEF through its RI improvement program. Tanganyika and Haut-Lomami signed a memorandum of understanding with BMGF managed by PATH, in which both the foundation and provinces contributed to a basket fund to finance operational activities for the Routine Immunization System Strengthening project.^22^^,^^23^ Additionally, these provinces benefited from additional support from the United Kingdom Department for International Development, World Bank, and the U.S. Agency for International Development.

A key feature of the Mashako Plan was the redistribution of existing funds allocated to support immunization services. Existing funding from Gavi, UNICEF, and BMGF was repurposed to fund Mashako Plan activities. The goal was to better use existing funds from donors rather than inject new funds to improve the system, thus piloting a more sustainable and cost-effective intervention. In the provinces that had a memorandum of understanding, funds that had previously been provided by Gavi to support immunization services were provided by BMGF through a basket fund. At the beginning of the agreement, BMGF provided 100% of the funds with the agreed-upon plan to decrease funds each subsequent year by 25% to reach 0% by year 5. At the same time, the province was expected to incrementally increase its funds each year.

A key feature of the Mashako Plan was the redistribution of existing funds allocated to support immunization services.

A member of the EPI stated that the Mashako Plan was an effective strategy for improving routine immunization and could contribute to achieving the 2030 immunization priorities in the country. Furthermore, a program manager from the WHO expressed optimism that the plan had the potential to transform the perception of routine immunization in DRC. The Minister of Health was one of the strongest advocates of innovative management approaches to address immunization issues. To successfully launch the Mashako Plan, maintaining support from the MOH and partners was critical.

The plan focused on implementing 5 key interventions for sustained immunization practices in the country:

1. Coordination: Ensure regular coordination by conducting performance reviews of immunization activities at the national and provincial levels.
2. Service delivery: Increase the number of completed vaccination sessions by at least 20% within 18 months of implementation.
3. Vaccine availability: Reduce stock-outs by 80% and ensure that 90% of the supervised health facilities have all vaccines and antigens each month.
4. Real-time monitoring: Conduct supervision to provide real-time monitoring and feedback on activities.
5. Evaluation: Implement regular stock-takes and an annual independent survey to evaluate performance.

### Intervention 1: Coordination

To support the immunization program, weekly Mashako Plan coordination committee meetings were held at the national and provincial levels to manage the funding, implement activities, identify challenges, and propose solutions. During national committee meetings, MOH officials and partners discussed technical issues with implementation. At the provincial and health zone level, coordination meetings were integrated into existing monthly health monitoring meetings to avoid increasing the management team’s administrative workload. Committee meetings increased transparency in funding, clarified implementation issues, and provided a venue to discuss challenges and identify solutions to improve coverage rates, decrease stock-outs, and provide effective supervision and support.

### Intervention 2: Service Delivery

The diagnostic review identified immunization service availability as a major constraint for increasing coverage, with 33% of health areas conducting less than 1 immunization session weekly and only 10% organizing more than 2 sessions weekly. The planned immunization sessions before the Mashako Plan were not related to the population served or catchment area and were below the WHO recommendations for service delivery.^24^ To standardize service delivery and provide simple minimum service guidelines, health areas were divided into 3 categories based on their population (low, average, and high), the immunization calendar, and the average number of children observed per immunization session. For each category, a minimum number of immunization sessions was recommended to improve frequency and regularity. Each health area in the 9 provinces was assigned a minimum number of immunization sessions to conduct every month:

- At least 4 immunization sessions per month for the low population areas (population <10,000)
- At least 8 immunization sessions per month for average health areas (population 10,000–15,000)
- At least 12 immunization sessions per month for highly populated health areas (population >15,000)

The targets provided were designed to be easily remembered by vaccinators and not conflict with facility plans if they chose to organize more than the minimum number of sessions. The estimates were based on the overall population numbers of the area, and thresholds were selected to make them easy to remember for nurses. These minimum targets were designed to be met without requiring significant additional resources.

### Intervention 3: Vaccine Availability and Cold Chain Functionality

The diagnostic review of the immunization system identified cold chain functionality and vaccine availability as major barriers to the reduction of future vaccine stock-outs. The Mashako Plan aimed to ensure that 90% of health facilities with a refrigerator had a security stock of all the antigens and consumables required to fully immunize a child every month.

To accomplish this goal, the first implementation activity focused on improving the regularity and quantity of vaccines delivered at each level. At the national level, instead of using only population-based estimates to determine the quantities of vaccine delivered to provinces, updated delivery schedules were generated to also account for the quarterly estimated needs compared to monthly needs, additional security stock levels, and transportation time. This change increased the number of vaccines delivered to provincial warehouses.

In addition, vaccine warehouses at each level were required to implement a more conservative threshold for vaccine requests and to respect security stock minimums. Managers were required to request vaccines before they reached the security stock threshold, typically when only 1 month of stock remained, instead of ordering at fixed times to maintain minimum stock levels all year. Instead of health facility nurses acquiring vaccines from the health zone, health zones were offered an additional stipend to deliver vaccines to health facilities. The change in vaccine direction aimed to provide regular vaccine delivery to health facilities. Health facilities also received additional funding to ensure adequate maintenance of the cold chain.

### Intervention 4: Real-Time Monitoring

An essential component of a high-functioning immunization system is the ability to monitor for challenges, which allows for quick problem identification and solution development. The Mashako Plan aimed to implement real-time monitoring of health facilities by routinely supervising health zones and health facilities. The EPI program worked with an external consulting and management agency, Acasus, to address data collection issues. Thematic groups at the national level were in charge of the development of indicators that were then selected based on the most important interventions that the leadership hoped to improve. During workshops, the government and partners agreed upon the calculation method; these methods were developed to be simple and to easily facilitate performance comparison between districts. A monitoring system was set up to collect data electronically at the health facility level using a smartphone app called Gestion PEV. Supervisors from each health zone management team were provided with smartphones, Internet credits, and training to conduct monthly supervisions of at least 1 health facility in each health area of each health zone. Mobile surveys were used to track activities at the health facility and health area level, verify improvements in supervision, and collect real-time monitoring data on vaccine indicators. The initial 9 provinces consist of 191 health zones and 3,456 health areas; therefore, every month, 3,456 supervision visits in health facilities were expected to be completed.

The Mashako Plan aimed to implement real-time monitoring of health facilities by routinely supervising health zones and health facilities.

During the monthly supervision of selected health facilities by a health zone manager, specific questions were asked about the quantity of each vaccine available and the number of vaccination sessions planned and carried out, and pictures were taken to document facility stock and operations during vaccination sessions. Additionally, the type and model of the available cold chain were noted, as well as its functionality and temperature range for the month. The surveys collected data on 6 indicators that were aggregated at the health zone level to evaluate overall performance:

- Percentage of health areas supervised (monitoring)
- Percentage of health areas performing the minimum number of immunization sessions (service delivery)
- Percentage of health areas with a functional refrigerator (vaccine availability)
- Percentage of health areas with sufficient vaccine availability (vaccine availability)
- Percentage of health areas that held their monitoring meeting (coordination)
- Percentage of health areas that submitted their immunization data in the DHIS2

**FIGURE 4:**
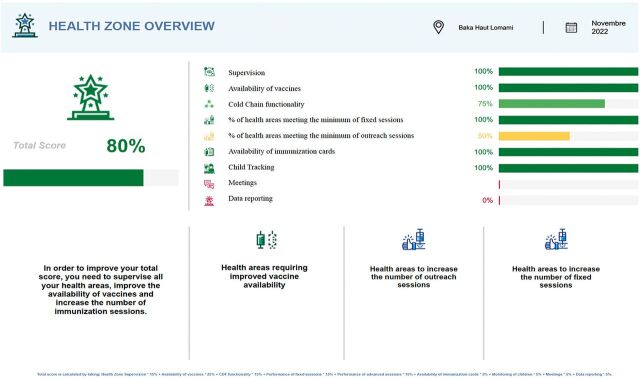
Sample Health Zone Scorecard Based on EPI Mobile Supervision Data Collected Monthly Abbreviation: EPI, Expanded Programme on Immunization.

At the end of each month, report cards were generated for the health zone, provincial, and national levels from the supervision reports submitted to the national EPI program (Figure 4). Each health zone was ranked by province for both its overall performance score and for each indicator. An overall score was calculated as a weighted average of the 6 indicators. Health zone performance was categorized into low, average, and high performing based on the score they received for each indicator. At the national level, the 9 provinces were ranked based on their score and ability to improve the tracked indicators. The scorecards were designed to show performance in a visually appealing way and to simplify the decision-making process by progressing from general information (e.g., overall score) to more granular data (e.g., performance by health area). The colors were chosen to match the generally universal standard of green as good performance and red as poor performance. These scorecards were provided monthly. At review meetings, the manager would provide average quarterly and annual performance estimates.

### Intervention 5: Assessment and Evaluation

Regular assessments and evaluations were a key intervention in the Mashako Plan policy; reviews were held biannually at the national level and quarterly at the provincial level. Quarterly reviews were organized at the provincial level to track implementation progress. During these reviews, the Mashako Plan committees presented the progress observed using the real-time monitoring system and discussed solutions to improve the indicator scores over the next quarter. These quarterly reviews were an opportunity to maintain momentum for change, and each province was able to recognize the best-performing health zone managers.

Quarterly reviews were an opportunity to maintain momentum of change and track implementation progress.

The biannual national reviews were chaired by the national Minister of Health to review overall progress in the 9 provinces. During these reviews, the 3 best-performing provinces and the 3 best-performing health zones received a certificate of recognition, signed by the Secretary General, for their exceptional contribution to RI in the DRC. These national reviews were a platform for provinces to share their best practices in hopes of improving their performance over the next 6 months.

To measure progress toward the 15-percentage point improvement goal for full immunization coverage, the Kinshasa School of Public Health (KSPH) at the University of Kinshasa conducted yearly independent vaccination coverage surveys (VCS). These surveys used rapid multi-cluster sampling methodology to obtain estimates at the health zone level that are compiled for provincial estimates, compared to the standard large surveys, including the MICS, which only provides provincial-level estimates. The surveys use mother or guardian interviews in selected households to record information about routine vaccinations and review vaccination cards when available. By October 2020, coverage surveys were completed in18 provinces, including the 9 initial Mashako Plan provinces.^25^

The Mashako Plan provides important insight into cost-effective measures that can be implemented to improve the RI system. After monitoring and evaluation of the RI system were strengthened by Mashako Plan activities, multiple data sources were used to assess changes in vaccination coverage over the implementation period. These data sources include the monthly supervision data and report cards from the Gestion PEV app, the KSPH health zone–level VCS, and the administrative data stored in DHIS2.

## EVALUATING THE IMPACT OF THE MASHAKO PLAN

To evaluate the impact of the Mashako Plan, we looked at the vaccine coverage estimates from the DHS 2013–2014 survey, the MICS 2017–2018 survey, the baseline VCS 2018–2019 survey, the VCS 2020 survey, and the VCS 2021–2022 survey.^9^^,^^10^^,^^25^^–^^27^ The VCS surveys conducted in 2018–2019 (5 provinces), 2020 (18 provinces), and 2021–2022 (26 provinces) were conducted using the same methodology. These 5 time points provide a general trend of the change in coverage between 2013–2014 and 2021–2022. The 2018–2019 baseline VCS survey was performed in only 5 of the 9 initial Mashako plan provinces due to operational constraints.^26^ The 2021–2022 VCS survey was conducted following the main impacts of the COVID-19 pandemic and system-wide nurse strikes that disrupted provision of immunization services in the DRC.^7^

While it is difficult to make direct comparisons due to methodological differences, including the lowest level at which estimates can be disaggregated (provincial–MICs vs. health zone–KSPH), these surveys still provide some sense of changes in overall performance between time points at the provincial level. Some Mashako Plan interventions, such as improvements in vaccine delivery from the national to provincial warehouses, would have benefited all provinces; however, this does not account for all gains made in non-Mashako Plan provinces.

The 2020 KSPH VCS indicated that there was a larger increase in coverage in the 9 Mashako Plan provinces than in the 9 non-Mashako Plan provinces when compared to other national surveys, such as the DHS and MICS. In the initial provinces, we generally see increases in DTP3/penta3 coverage over time. Data for the 9 Mashako provinces shows that DTP3/penta3 coverage in the 2020 survey increased by 12.7 percentage points compared to the DHS 2013–2014 and by 28.4 percentage points compared to the MICS 2017–2018.^10^^,^^11^^,^^25^ The drop observed in 2021–2022 is likely due in part to the global impacts of COVID-19 and the health care worker strikes in the DRC that resulted in the temporary closure of health facilities in the DRC and subsequent reduction of immunization services provided in the DRC (Table 2).^27^ We see similar trends for full immunization coverage (Table 3).

In the 5 provinces of Kinshasa, Kwilu, Kasai, Haut Lomami, and Tanganyika, the same VCS methodology was used in 2018–2019, 2020, and 2021–2022.^25^^–^^27^ For these provinces, DTP3/penta3 coverage in the 2020 survey increased by 16.6 percentage points when compared to the baseline 2018–2019 survey, and all 5 provinces showed improvement in DTP3/penta3 coverage. Two of the 5 provinces continued that trajectory in the 2021–2022 VCS survey, while 3 of the provinces saw a drop in penta3 coverage, which may be due to a combination of the health care worker strikes in these provinces and the impact of COVID-19. Penta3 coverage remained higher in 2021–2022 when compared to the baseline (+11.1 percentage points) for the 5 provinces.

**FIGURE 5:**
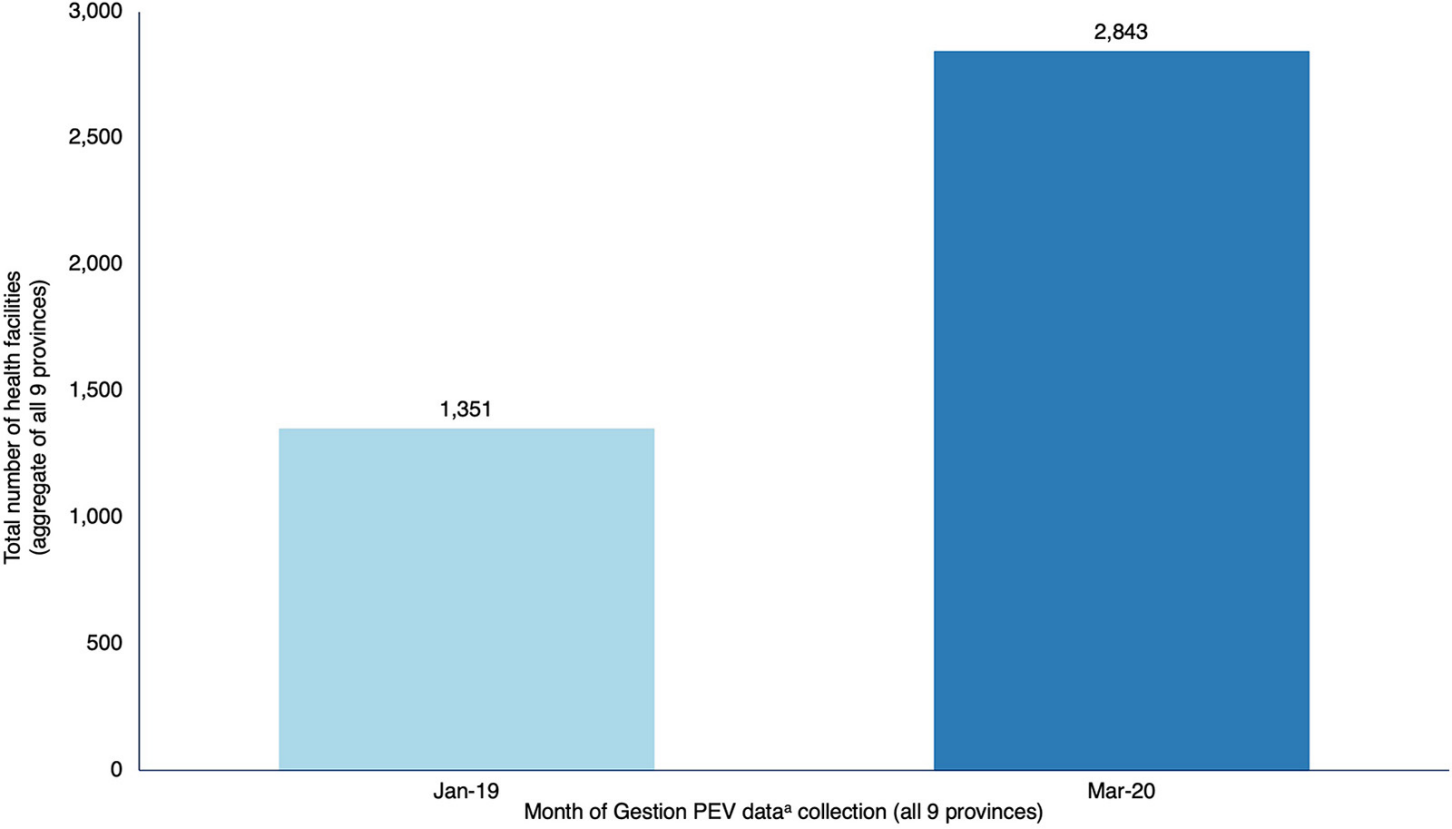
Number of Health Facilities Supervised in Initial Mashako Plan Provinces Abbreviation: EPI, Expanded Programme on Immunization. ^a^Gestion PEV data was collected through the real-time EPI mobile supervisory system developed by Acasus.

Several process indicators point to the same level of improvements in outputs in the system. The Gestion PEV supervisory system provided an estimate of changes in performance indicators over the implementation period. In January 2019, only 1,400 health areas were supervised through the Gestion PEV app. By January 2020, more than 3,400 (∼80%) health areas were routinely supervised each month by a health zone manager—a 30% increase (Figure 5).

**FIGURE 6:**
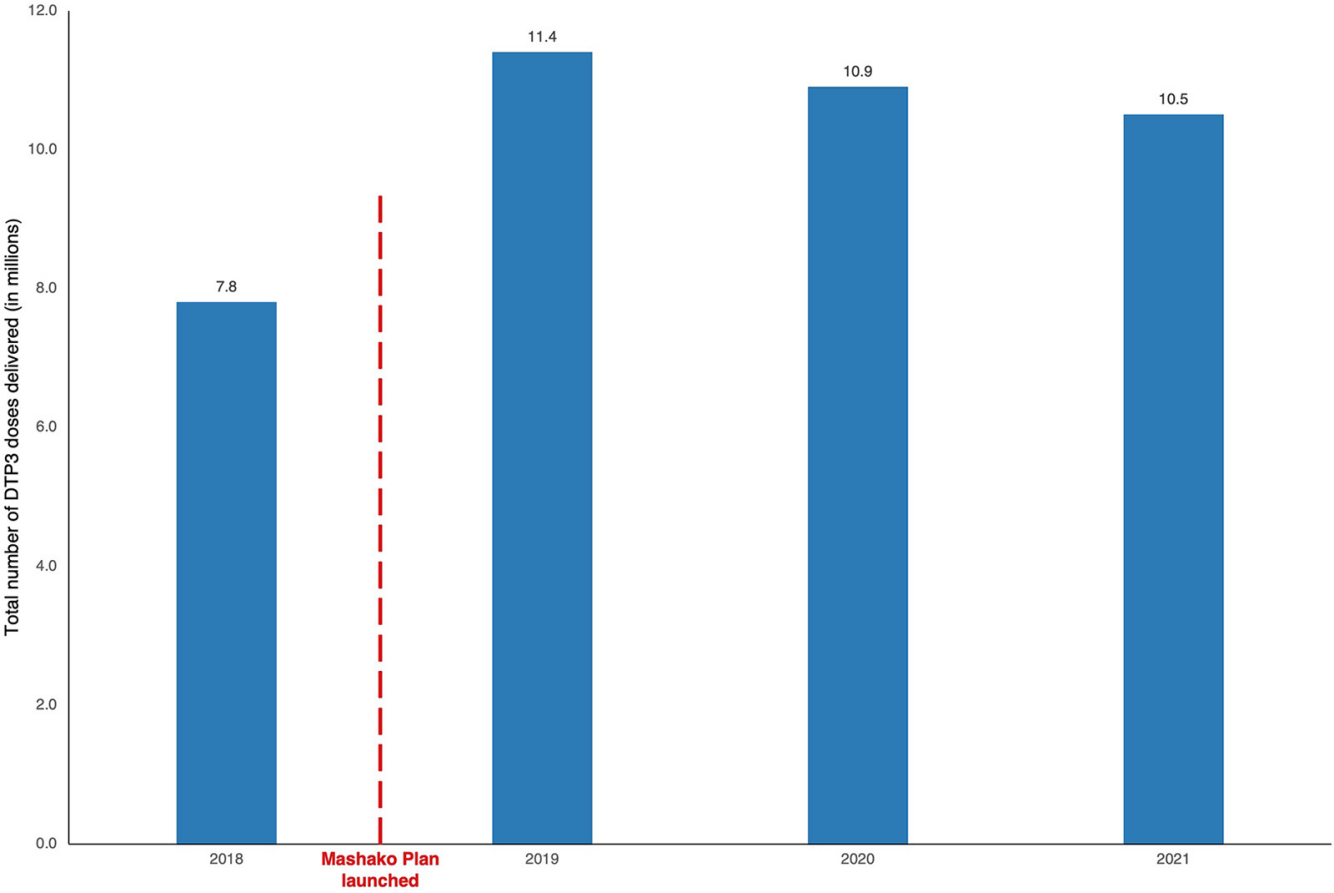
Annual Pentavalent Deliveries in Million Doses^a^ From the National Warehouse to All Provinces, Nationally Abbreviation: DTP3, diphtheria, tetanus, pertussis. ^a^ Pentavalent vaccine dose estimates from the National Stock Management Tool data.

In March 2020, 83% of supervised health areas were compliant with the minimum immunization session targets, and 82% had good vaccine availability, increases of 50% and 19%, respectively, from January 2019 (Table 4). Additionally, data from the National Stock Management Tool show increased deliveries of penta3 vaccine to the provinces by 46% in 2019, 39% in 2020, and 33% in 2021 compared to 2018, after the implementation of the Mashako Plan (Figure 6).

**TABLE 4.** Changes in Selected Indicators During Mashako Plan Implementation, Democratic Republic of the Congo^a^

**Indicator**	**January 2019, %**	**March** **2020, %**	**Difference, Percentage Points**
Health areas supervised	30	80	+50
Health areas that organize weekly vaccination sessions	50	62	+12
Cold chain with good functionality	73	94	+21
Vaccine availability	19	73	+54

a Source: Gestion PEV app.

Administrative data obtained from DHIS2 show similar improvements in service delivery when compared to supervisory visits. Between June 2018 and March 2020, the percentage of health facilities reporting less than 4 immunization sessions per month decreased from 33% to 12%, and the percentage reporting more than 8 immunizations sessions per month increased from 10% to 38% in the 9 Mashako Plan provinces (Table 5). Additionally, these provinces added more than 8,000 monthly immunization sessions after a year of implementation, a 53% increase in the 18-month period. This meant that, on average, each health area added 2.5 immunization sessions per month. In comparison, non-Mashako Plan provinces increased the number of immunization sessions by only 5% over the same period.

**TABLE 5.** Changes in Reported Monthly Immunization Sessions^a^ in Health Areas During Mashako Plan Implementation, Democratic Republic of the Congo

**Health Area Vaccination Sessions Per Month**	**June 2018**	**March 2020**
	**No. (%) (N=2,881)**	**No. (%) (N=2,844)**
<4	951 (33)	341 (12)
4–8	1,642 (57)	1,422 (50)
>8	2,88 (10)	1,081 (38)

a Reported immunization sessions are a component of service delivery with reported administrative data obtained from the DHIS2 in both June 2018 and March 2020.

While the specific interventions of the Mashako Plan were ongoing, the government and donors were implementing other health system strengthening interventions. Gavi’s Cold Chain Equipment Optimization Platform, with financial support from the World Bank, enabled the installation of over 4,000 solar refrigerators in health facilities in all provinces, improving accessibility to immunization services and vaccine storage. This increase in cold-chain capability increased points of service for immunizations by providing long-lasting, functional cold-chain capability. In July 2019, the MOH, in conjunction with BMGF and PATH, coordinated and hosted a presidential forum as a first review of the Mashako Plan implementation. During the forum, representatives from the MOH, EPI, participating province governors, and partners reviewed progress and committed to continuing the efforts to promote vaccination. The President formally committed to full government funding of vaccine purchase commitments in partnership with Gavi, including the amounts for traditional vaccines. The governors committed to actively supporting immunization in their provinces. The country improved its funding for traditional vaccines, as well as Gavi co-financing, in 2019, 2020, and 2021.

## DISCUSSION

The Mashako Plan is similar to plans that have been implemented in other LMICs, such as the Mission Indradhanush in India and the Reaching Every District Using Quality Improvement approach in Ethiopia.^28^^,^^29^ These programs attempted to strengthen national RI systems and, like the Mashako Plan, used local community engagement to improve vaccination rates, especially working to identify unimmunized children. Unlike the Mashako Plan, these strategies did not implement additional national strategies to improve political will to support immunization programming financially or address other systemic issues, such as cold chain functionality, that impact the success of a RI system. However, they did incorporate serosurveys to validate coverage estimates in support of RI programming.^28^ The Mission Indradhanush program in India achieved minimal success with a recommendation to adapt the program from purely a vaccine delivery system to a disease control model. Conversely, the program in Ethiopia led to significant improvements in vaccine coverage outcomes that were validated with the use of serosurveys.^28^^,^^29^

The Mashako Plan was initially successful due to a strategy that focused on 5 key challenges associated with poor RI: coordination, service delivery, vaccine availability, real-time monitoring, and evaluation. This strategy of RI system revitalization has the potential to be an important method of cost-effective system strengthening of RI systems, both when expanded to other DRC provinces (Mashako Plan 2.0) and beyond to other LMICs. The plan has succeeded because of the strong support of subnational government leaders and the development of small achievable goals that increased the accessibility and availability of vaccination services at health facilities.

The Mashako Plan has succeeded through strong subnational government support and the development of small achievable goals.

Several key lessons from the initial period of Mashako Plan implementation were identified, all of which pointed to the tremendous impact of a strategy that focused on the most critical EPI issues affecting accessibility in the RI system. Throughout the initial rollout of the Mashako Plan, extensive advocacy and coordination were necessary to establish government and partner agreement on a common platform to review progress. The EPI program led the design and implementation of the project and created a steering committee to coordinate all stakeholder collaboration. There was consensus on the need for strong coordination efforts and the importance of shared responsibility between all stakeholders, including national leadership, international stakeholders, and local health care counterparts. Innovative use of technology to support immunization activities, such as a mobile supervision app for systematic supervision, was critical to the plan’s rollout and success. The real-time monitoring provided a platform for quick evaluation and rapid corrective actions regarding supervision, stock management, and immunization sessions, which enhanced the accountability and transparency necessary for success.

Development of measurable indicators with frequent real-time monitoring and feedback allowed for buy-in and built trust in the system. Rapid improvements were possible in a complex setting through strong partnerships and engagement with national and local partners and simple evidence-based decision-making. Consistent messaging and feedback between the subnational and national levels built engagement and trust from the health facility up to the national level.

A finite number of interventions were chosen to ease monitoring and accountability and increase the chances of success. The interventions were designed to be simple to understand and implement (i.e., 1 immunization session per week).

While many of the key indicators showed quick progress, additional efforts are still necessary to reach the full program goals required to improve immunization outcomes. In part, this is achievable by adapting the interventions as the system improves and setting realistic, attainable targets to inspire confidence in the project.

### Challenges

Critical challenges to the implementation of the Mashako Plan included the delay of funding to support activities and disagreement on how to effectively allocate resources. In part, funding was delayed due to late or missing justifications. Transparency of resource allocation at the subnational level was not yet achieved at the end of the 18-month period.

Critical challenges to implementation included the delay of funding to support activities and disagreement on how to effectively allocate resources.

Availability of vaccines was another challenge. Real-time monitoring indicated that at least half of the health facilities ran out of at least 1 antigen or consumable at some point during the 18-month implementation period. On average, 15% of children that were present at an immunization session didn’t receive 1 of the antigens on schedule due to a stock-out. While revitalization activities can improve vaccine management at the local level, they are still impacted by national stock-outs related to funding issues or global supply chain delays. For example, in early 2019, there was a stock-out of bacillus Calmette-Guérin vaccine due to late payments from the government for the purchase of vaccines. In addition to vaccine availability, the plan did not directly target infrastructure limitations, such as cold chain saturation in health facilities, availability of transportation equipment, access to remote areas, and insecurity issues. Vaccine deliveries improved substantially at national and provincial levels, but the gains were much lower at the zonal and heath area levels. Unfortunately, even if a stipend was provided to facilitate vaccine distribution, there was no system to ensure that the task of vaccine delivery to the health facility was accomplished. Nurses were still expected to pick up vaccines if they were not delivered, and while management did improve, it did not meet the expected level of improvement that was predicted at the start of plan implementation. While this plan addressed many critical issues, it did not address all areas of the immunization system as it did not impact vaccine demand, disease surveillance, or human resource challenges (e.g., pay, staff ability, and staff numbers). The Mashako Plan provided training and guidelines on the distribution of vaccination cards to mothers. The national level also improved the distribution of cards to the provinces. There were also challenges related to physical access barriers in some provinces, such as Ituri, due to the ongoing conflict in the eastern part of the DRC.

### Limitations

There are several limitations to the data collected and analyzed in the presentation of the Mashako Plan. Coverage estimates from surveys performed before, during, and after the initial implementation period were used as 1 of the data points to understand the plan’s impact. The DHS 2013–2014 and MICS 2017–2018 survey results were used as an estimate of vaccination coverage rates before policy implementation; however, the methodology of the MICS survey differed from that of the VCS surveys. The estimates from the MICS 2017–2018 were not used for the WHO/UNICEF Estimates of National Immunization Coverage due to inconsistencies in the caregiver recall. The 5 surveys presented provide a general trend for immunization coverage, in addition to the changes observed in process indicators (service delivery, logistics, and supervision). Additionally, the methodology for the VCS surveys performed in country was approved for use in the WHO/UNICEF Estimates of National Immunization Coverage. These surveys are now run annually and will continue to provide coverage estimates in the following years, which will be especially helpful to the zero-dose agenda. In addition, our estimates use the DRC standard estimate of 3% population growth per year.^30^

## CONCLUSIONS

The Mashako Plan succeeded in increasing RI coverage and key service delivery indicators over the 18-month implementation period, contributing to nearly 360,000 additional children fully vaccinated. The strong commitment of MOH staff at all levels combined with partners’ involvement provided pressure on the whole system to improve. The simple set of interventions and indicators focused the energy of managers on discrete actions to improve outcomes.

*[The Mashako Plan] has allowed us to take our discussion centers out of the vicious circle (epidemics and campaigns), and it has also allowed us to make changes in mentality in the monitoring of activities in the way we supervise.* —Interview with Gavi representative

To strengthen RI, it is important to focus not only on the goal of increased immunizations but also on the system that supports (or hinders) that goal. In February 2020, the MOH planned the extension of the Mashako Plan to all provinces of the country by the end of 2020 and began reviewing indicators that emphasize outreach and vaccine demand generation. However, the COVID-19 pandemic and other implementation challenges due to the massive scale-up delayed the rollout to new provinces. By the end of 2020, 18 provinces had been integrated into the Mashako Plan. In January 2022, indicators were updated in the Mashako Plan 2.0 to account for demand and increase focus on outreach.

Financing, personnel, and transport challenges should be evaluated and potential adaptations made during rollout of the plan to remaining provinces or when it is used as a model for strengthening RI systems in other countries. Given the external commitments and strong collaboration that would be required to replicate this strategy in other LMICs, its long-term sustainability and applicability outside of the DRC should be evaluated in more detail. Overall, the Mashako Plan demonstrates that simple and measurable changes to the immunization system at all levels can quickly improve immunization coverage; however, sustainability of improvements cannot be measured in only 18 months. Further exploration of the results will be necessary to determine the plan’s long-term impact and generate all-level engagement for sustainable success.

## Notes

**Peer Reviewed**

**First published online:** April 13, 2023.
